# Rethinking our future: Describing and enhancing the impacts of dissemination and implementation science for cancer prevention and control

**DOI:** 10.1017/cts.2024.587

**Published:** 2024-10-10

**Authors:** Vianca Cuevas Soulette, Karen M. Emmons, Douglas A. Luke, Peg Allen, Bobbi J. Carothers, Ross C. Brownson

**Affiliations:** 1 Prevention Research Center, Brown School, Washington University in St. Louis., St. Louis. MI, USA; 2 Department of Surgery (Division of Public Health Sciences) and Alvin J. Siteman Cancer Center, Washington University School of Medicine, St. Louis. MI, USA; 3 Department of Social and Behavioral Science, Harvard T.H. Chan School of Public Health, Boston, MA, USA; 4 Center for Public Health Systems Science, Brown School, Washington University in St. Louis., St. Louis. MI, USA

**Keywords:** Cancer control, dissemination and implementation research, impact, translational benefits, translational science

## Abstract

**Background:**

Researchers generally do an excellent job tracking the scientific impacts of their scholarship in ways that are relevant for academia (e.g., publications, grants) but too often neglect to focus on broader impacts on population health and equity. The National Cancer Institute’s Implementation Science Centers in Cancer Control (ISC3) includes 7 P50 Centers that are interested in broad measures of impact. We provide an overview of the approach underway within the ISC3 consortium to identify health and social impacts.

**Methods:**

ISC3 adapted and applied the Translational Science Benefits Model (TSBM) to identify the impact on the discipline of D&I science and to consider dissemination and implementation (D&I) impacts in the four original TSBM domains: (1) clinical; (2) community; (3) economic; and (4) policy. To collect data from all Centers, we: (1) co-developed a set of detailed impact indicators with examples; (2) created a data collection template; and (3) summarized the impact data from each center.

**Results:**

Based on data from 48 ISC3 pilot studies, cores, or consortium activities, we identified 84 distinct benefits. The most common impacts were shown for implementation science (43%), community (28%), and clinical (18%). Frequent audiences included primary care providers, public health practitioners, and community partners. ISC3 members highlighted the need for product feedback, and storytelling assistance to advance impact.

**Conclusions:**

The ISC3 consortium is using a participatory approach to successfully apply the TSBM, thus seeking to maximize the real-world impacts of D&I science. The D&I field needs to prioritize ways to more fully document and communicate societal impacts.

## Introduction

Researchers, including those in dissemination and implementation (D&I) science, contribute significantly to the generation of collective knowledge and excel in tracking the scientific impact of their scholarship through metrics relevant to academia, such as publications and grants. However, it is important to remember that the ultimate aim of our work is to benefit individuals and communities, and these traditional academic metrics have limited utility for demonstrating broader impacts on population health and equity. The public has increasingly called for academic institutions to justify their research expenditures [1]. These trends highlight the obligation that researchers, particularly those funded by public money, must demonstrate their accountability and the value derived from their research investments [2]. Simultaneously, funding bodies are eager to display the positive impacts of their research investments [3]. This urgency is compounded by a growing emphasis on minimizing waste in research processes [4]. Furthermore, there is a growing directive for researchers in the field of D&I science to develop and refine metrics that accurately measure equity and the impact of their research [5]. Given these factors, it is increasingly important for researchers to document and strategically communicate the impacts of their research, ensuring that the benefits are clear and tangible [6–8].

Several frameworks have been developed to assess broader impacts on practice and policy (Table 1). Among these, the Research Excellence Framework (REF), primarily used to evaluate academic institutions in the UK, assesses the impact of academic research on various domains such as the economy, society, culture, public policy, health, environment, and quality of life [9]. The strengths of the REF include its comprehensive nature and the positive correlations between impact scores and overall research quality [10]. However, the REF can be time-consuming and labor-intensive [11–15]. As discussed, the REF is primarily used to measure institutional-level impact, but other frameworks exist to measure research programs or projects. One is the Payback Framework, which offers a holistic approach to evaluating the impact of health services research [16–20]. Nevertheless, it requires significant resources, including detailed interviews and extensive analysis [21,22]. Another approach involves monetization models, which quantify the economic returns of research investments using metrics like cost savings or quality-adjusted life years (QALYs). This approach assesses the economic impacts but relies on simplifying assumptions about the time lag between research and its impacts and the proportion of benefits attributable to the research [23–26]. A hybrid method is The Framework to Assess the Impact of Translational Health Research (FAIT), which combines a modified Payback method, economic analysis, and narrative approaches to assess the research impact comprehensively [27]. It uses qualitative and quantitative methods, emphasizing narrative creation, and has been used in low- to middle-income countries [28]. However, FAIT’s retrospective application limits end-user perspectives and assumes a linear, sequential process, which does not accurately represent research translation [28,29]. For those interested in learning about each model’s domains, uses, benefits, and strengths, please refer to Table 1. For this paper, we will focus on the Translational Science Benefits Model (TSBM).

**Table 1.** Selected frameworks to document impact

Name of the framework	Country	Domains measured	Use of the framework	Strengths	Limitations
Research Excellence Framework (REF)	UK	Economy, society, culture, public policy, health, environment, and quality of life.	Evaluation of Academic Institutions’ Impact: The REF influences the allocation of around £2 billion in annual government funding for university research, playing a pivotal role in shaping research priorities and driving quality.	Ensures accountability for public investment in research; provides a comprehensive assessment of research quality. Positive correlations have been found between impact scores and overall research quality.	Time-consuming and labor-intensive preparation; assesses impact at the institutional level, not at the level of specific scientific initiatives.
Payback Framework	UK	New knowledge, future research, policy and product development, health sector advantages, and economic benefits beyond health.	Holistic approach to evaluate health services research impacts.	Structured Evaluation: multi-dimensional benefits, research utilization, evaluation of diverse research types, economic and societal impacts.	Requires significant resources for implementation, including detailed researcher interviews and extensive analysis; focuses on individual projects, potentially overlooking cumulative or synergistic impacts.
Monetization Models in Health Research	Various	Quantifies economic returns of research investments using metrics like cost savings and quality-adjusted life years (QALYs).	Usage is diverse, considering a range of costs and benefits, including health-related outcomes and broader economic impacts	Quantifies economic impacts, helping to understand costs associated with those impacts.	Relies on simplifying assumptions; subjectivity in quantitative estimates; challenges in attribution and methodological debates; complexity in balancing monetized and non-monetized impacts.
Framework to Assess the Impact of Translational Health Research (FAIT)	Various	Knowledge creation, health services, and policy impacts. The economic component primarily involves cost-benefit analysis. The narrative part details the journey of research translation and impact creation.	Combines modified Payback method, economic analysis, and narrative approaches to assess research impact, useful in diverse research environments including low- and middle-income countries.	Mixes qualitative and quantitative methods; emphasizes narrative creation.	Retrospective application limits the incorporation of the end-user perspective and assumes a linear and sequential research translation process.

In the USA, the TSBM was designed to evaluate the health and societal impacts that originate from translational science and was first used as an impact evaluation framework within the Clinical and Translational Science Awards (CTSA) program [30]. This model extends traditional research metrics that primarily track scientific progress (e.g., grants, publications, presentations). It takes an inclusive approach, focusing on identifying and measuring the direct impact of translational science on health outcomes in clinical settings and broader communities. The TSBM combines the latest advancements in creating frameworks and logic models specific to translational research [31]. This results in a comprehensive method, allowing institutions or individual researchers to assess the real-world impacts of their clinical and translational research. The model lists 30 specific benefits associated with clinical and translational science, organizing them into four particular benefits categories: clinical, community, economic, and policy [30]. Unlike several other frameworks, the TSBM was explicitly designed for translational science purposes. The TSBM is user-friendly, featuring an online toolkit with editable documents such as the Roadmap to Impact, Impact Tracker, Impact Profile, Case Study Builder, and Dissemination Planner. These resources are available online to facilitate impact assessment or to adapt for a research project, including a web tool that helps develop case studies. The TSBM designers have also recently begun to create a community-engaged approach to integrate health equity into the model, and those updates will be released soon. Each model has its strengths and limitations. Whether the goal is to evaluate an academic department, a research program, or a specific project or to conduct a comprehensive monetization analysis, it is essential to consider the tradeoffs among each of the tools mentioned above and determine which one might best fit the needs of a specific project.

This paper uses the TSBM framework to measure the collective impact of the Implementation Science Centers in Cancer Control (ISC3), a consortium of seven research centers devoted to cancer control and prevention funded by the Cancer Moonshot initiative at the National Cancer Institute [32]. The ISC3 includes Harvard T.H. Chan School of Public Health, Oregon Health and Science University, University of Colorado School of Medicine, University of Washington, Wake Forest School of Medicine, Washington University in St Louis, and the University of Pennsylvania [33–39]. By sharing the impact evaluation approach utilized by the ISC3, we aim to provide examples of how to document impact using the TSBM.

## Materials and methods

### Participants

All seven ISC3 centers participated, each with a unique focus on implementation science for cancer control and prevention. The Harvard T.H. Chan School of Public Health combines health equity with implementation science to create low-burden implementation tools in community health center settings [33]. Oregon Health and Science University targets cancer screening and prevention for underserved groups [34]. The University of Colorado emphasizes pragmatic approaches in rural care for cancer prevention—the University of Washington pioneers in refining evidence-based cancer control methods [36]. Wake Forest School of Medicine uses technology for real-time evaluations of cancer control processes [37]. Washington University in St Louis focuses on rapid-cycle research and systems science to address cancer disparities [38]. The University of Pennsylvania employs behavioral economics to speed up the adoption of equitable, evidence-based cancer care practices [39].

We executed a collaborative approach to distribute the data collection tool. Each center director could use a convenience sample to select at least two to three projects or units for TSBM impact assessment and data collection. Initially, leaders from several centers discussed the feasibility of the initiative and worked to secure buy-in from other directors, ensuring a unified and supportive approach across the consortium. Following these preliminary discussions, an email and meeting was held to discuss the data collection process. This communication underscored the necessity of capturing a broad spectrum of impacts using the Translational Science Benefits Model as a guiding framework. The email provided a template of the data collection process initially pilot-tested at Washinton University ISC3, highlighting the practicality and minimal time commitment needed to fill out the template. Additionally, we offered support through a planned webinar to clarify the process and address any queries, ensuring that each center could efficiently contribute two to three projects or units. This structured approach and proactive engagement with center leadership across the consortium were designed to enhance the accuracy and comprehensiveness of our impact documentation, emphasizing both individual and collective achievements across the centers.

In selecting a practical framework for measuring impact, we used the TSBM to highlight the impact of our research beyond academic publications. The TSBM was chosen because of its comprehensive list of domains and user-friendly online tools, which made it easier to adapt for our impact assessment purposes. This decision was motivated by our desire to demonstrate our impact to funders and to convert research findings into success stories for broader dissemination to diverse audiences. The ISC3 consortium leadership adopted a participatory development approach to formulate a set of TSBM Indicators specifically tailored for Implementation Science (Table 2).

**Table 2.** Impact indicators list for the implementation science center for cancer control consortium

Implementation Science Center for Cancer Control Impact Indicators
1. Implementation Science Domain
**1a- Implementation Science Methods and Measures**
*Measures Development* Developed new measures of implementation determinants, processes, or outcomes Developed new methods for implementation strategy selection and optimization, or for identifying and prioritizing implementation determinants Developed new methods and toolkits for identifying and applying Theories, Models, and Frameworks (TMFs) focused on health equity Conducted comprehensive summaries of existing methods and made recommendations for improvements Developed new engagement or co-creation strategies Developed pragmatic costing tools to inform decision makers and Implementation Science (IS) researchers Developed methods for assessing implementation and setting context Identified gaps in the literature *Use of Rapid Cycle/Data Collection Strategies* Rapid needs assessment Used rapid cycle testing designs *Adaptation* Developed pragmatic, low burden approaches to measuring adaptation, fidelity, and implementation cost Technological tools for tracking adaptations in clinical and community settings Function (vs form) focused fidelity scales that are easy to administer in clinical and community settings Developed or adapted an implementation process or strategy with an explicit focus on health equity Developed methods for examining clinical/community partner data in new ways/formats that supports their work
***1b. Capacity-Building***
*Building partner/practitioner research capacity* Partner led or participated on grants, publications, presentations Developed partner’s own research infrastructure (e.g., pre- and post-award management, Facilities and Administrative Costs (F&A), Data Universal Numbering System (DUNS), biosketches) Developed partner skills in implementation processes Developed practitioner toolkits for integrating equity and/or costing into implementation science Developed tools to encourage the iterative use of IS frameworks to plan for, make midcourse adaptations during, and sustain evidence-based interventions (EBIs) in practice Developed tools to encourage the iterative use of IS frameworks by partners *Engagement* Developed strategy for return of results to research partners and beyond; strategy is preferred by partner, relevant and actionable Partner included in selection of pilot grants Increased diversity of engaged partners *Build IS research capacity* Included early investigators, trainees Increased diversity of investigator teams Increased skills of mentors Increased skills of early investigators and trainees at all levels Developed investigator toolkits for integrating equity into IS Extended IS efforts in the context of the partnership to new content areas in cancer control (e.g., climate change) Developed/Refined tools to aid in the planning of IS projects, selection, combination, adaptation, use and assessment of IS TMFs
2.Clinical Domains
**2a. Procedures/Guidelines**
*Diagnostic, investigative, or therapeutic procedures* Help i-Lab partners to address guidelines changes or new discoveries in terms of additional clinical services needed by patients Documented the impact of efforts to help partners address guideline recommendations Developed guidelines for navigators, community health workers, peer support team members, etc., on how to deliver health interventions for specific populations Developed treatment or implementation manuals Developed and used methods that have changed how research can be done within i-Lab partner’s organization, and specifically within health systems (e.g., processes or methods (e.g., use of artificial intelligence(AI)/machine learning (ML). An i-lab’s work is used to develop United States Preventive Services Task Force (USPSTF) or Community Preventive Services Task Force (CPSTF) guidelines Reduced use of low value clinical practices overall, and among patient who experience inequities
**2b. Tools and Products**
*Equipment & supplies, software technologies* Used technology to increase implementation of EBIs and/or to reduce inequities (might either increase use of EBI or enhance reach) Developed/refined and implemented software infrastructure to deliver test results related to EBI *Patient tracking tools* Used technology to track patients who have care gaps *Clinic-level tracking tools* Used technology to track the update and sustainment of EBIs and/or implementation strategies *Workflows* Workflow development related to EBI, or other support to help with implementation of high value cancer prevention and control Activities/services
3. Community
**3a. Health care delivery, health activities & products**
*Community health services* Increased testing/screening in target setting Created linkages between clinics to share strategies for use of EBIs; linkages lead to improved uptake of EBIs Developed and/or tested different training strategies Provided training to clinical service providers Increased access to care (e.g., telehealth) overall, and among groups experiencing inequities; documented impact on access and inequities Improved health care delivery by training peer supporters; reduced inequities. Number of clinics reached through dissemination or direct implementation of EBIs, in reference to “denominator” of those that could be impacted or characteristics of those who participated Reduced use of low value practices overall, and among patient who experience inequities Sustained use of EBIs and/or implementation strategies in partner organizations that participated in pilots Developed new implementation support resources for clinics *Health education and community resources* Developed new patient health education resources (ideally culturally and linguistically appropriate) Developed and tested messages related to increasing use of EBI overall, and specific to populations experiencing inequities Created a How-To guide for implementation in primary care clinics and community settings Developed a website with EBI-focused health education resources Developed apps to increase engagement in cancer prevention behaviors (e.g., physical activity or health diet) Developed new networks for prevention activities
**3b. Health Care Characteristics**
*Health care accessibility, health care delivery and quality* Tested interventions to improve access, delivery, quality of cancer screening and prevention (e.g., tested transportation options to cancer screening center) Developed new delivery channels (e.g., mobile services, telehealth); documented improvement in equity or at least no worsening Identified implementation strategies that are most effective at increasing the uptake of the EBI and at reducing inequities *Life expectancy* Reductions in stigma (through tested measures/Evidence-Based Interventions) increased engagement in care and may have improved health outcomes. Rapid, widespread infectious disease testing of large populations allowed people to return to work, school, and their daily activities safely, and may have improved quality of life. Reduced prevalence of key risk factors
**3c. Health Promotion**
*Disease prevention* Increased uptake of EBI leads to improved markers of health and/or reduced inequities Increased access to treatment for preventable diseases, overall and/or by groups experiencing inequities Reduced use of low value preventive practices overall and/or by groups experiencing inequities *Public health practices* Increased uptake of preventive services Increased infectious disease testing capacity and in turn disease surveillance Supported partner program evaluation via application of IS methods.
4. Economic
***4a. Commercial Products***
*License agreements* Developed Creative Commons License for materials *Application for/receipt commercial or partner-focused grants* Small Business Innovation Research (SBIR)/Small Business Technology Transfer (STTR) grant Partner-focused infrastructure, programmatic, or evaluation (non-research) funding *Development of nonprofit or commercial entities Patents Development of business cases*
**4b. Financial Savings and Benefits**
*Cost-effectiveness* Determined the most cost-effective way to implement EBI in health systems Determined the most cost-effective way to reduce inequities on an EBI *Cost savings* Reduced lifetime costs associated with a disease prevented by implementation of an EBI *Societal & financial cost of illness* Adopted policies to prevent and reduce causes of poor health that could lead to cost savings for society Adopted policies to reduce use of low value care Reduced societal costs of the target disease such as lost work productivity with EBI Quantified how insurance coverage affecting use of an EBI allows for modeling of cost savings and disease incidence reduction Documented costs to practices to improve partner decision-making about implementation options
5. Policy
**5a. Advisory Activities**
*Committee participation* Presented on target work at National Academy of Medicine (NAM) Served on NAM committee or professional board to evaluate uptake of EBI Presented target work to local, state, or national health/cancer committees for consideration in organizational and policy planning *Expert testimony* Provided expert consultation on evidence-based policy approaches to state/federal legislators *Scientific research reports* Included work in National Academy of Medicine (NAM) report. Included I-Lab’s work in a local or state-level report providing evidence-based recommendations
**5b. Policies and Legislation**
*Legislation, Policies* Work that increased legislators’ engagement with EBIs and relevant policies may lead to legislation and/or policy adoption States adopted legislation requiring clinician training on EBI Policies adopted based on EBIs improved equity *Standards* Work that leads to adoption of industry standards related to EBI Adoption of industry standards related to EBI likely to improve equity
**5c. Public Health Practices, guidelines, strategic plans (small p policy)**
*General* Assisted, contributed, and informed development of a local, tribal, state, federal, international strategic plan Contributed to a mandated Community Health Needs Assessment or Community Health Improvement Plan Helped an organization to use Evidence-Based Cancer Control Programs (EBCCP) or the Community Guide for selection of evidence-based cancer control programs *Federal Scientific Activities/Providing leadership in the field to promote IS* Disseminated information or served as IS Advisor on National Institutes of Health (NIH) Committees/Boards as part of the funded project Findings led to considerations included in Healthy People White House (WH) presentations (e.g., WH Meeting on Hunger and Nutrition) Informed NIH/National Cancer Institute (NCI) research agendas

### Clinical

Clinical domain efforts are centered on creating and updating procedures, guidelines, and tools to improve health outcomes. This includes developing new guidelines and manuals for health interventions, leveraging technology for patient tracking, and improving clinic workflows. The focus is also on reducing low-value practices and integrating advanced methods like AI to optimize health system research and implementation.

### Community

Community-focused indicators revolve around enhancing healthcare delivery, health activities, and products. Key activities include increasing testing/screening, developing training strategies, improving access to care like telehealth, and reducing health inequities. It also involves developing health education resources, creating implementation guides, and establishing networks for prevention activities aimed at better community health outcomes.

### Economic

The economic aspect involves developing commercial products and analyzing financial savings and benefits. This includes securing licenses, grants, and patents and developing business cases for health interventions. A significant focus is on determining cost-effectiveness, reducing disease-associated costs, and evaluating the societal and financial impacts of illness, emphasizing policy and practice improvements for better health economics.

### Policy

Policy-related indicators include advisory activities, legislation development, and public health practices. This involves contributing to national committees, providing expert testimony, and influencing public health reports. This field aims to foster legislation and policy adoption based on evidence-based interventions (EBIs), set industry standards, and advise on strategic health plans to integrate EBIs into broader health policies and practices.

### Addition of an implementation science domain: TSBM adaptation

**Figure 1.**
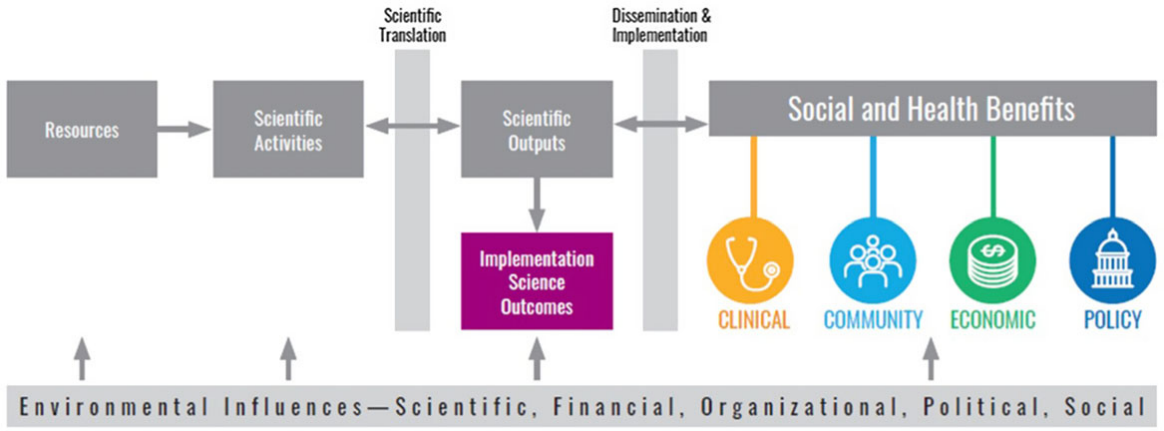
Adapted translational science benefits model.

We adapted the TSBM to align with the unique contributions of implementation science (Figure 1). This adaptation entails the introduction of a novel Disciplinary Outcomes domain within the TSBM, designed to allow assessment of the disciplinary impact of implementation science. This specific domain concentrates on developing and refining methods and measures in implementation science. It includes creating innovative tools for implementation strategy selection and capacity building, tools for intervention adaptation decision-making, tools to ensure end-user engagement in design phases, and more. Implementation science strategies refer to the systematic methods and approaches utilized to promote the adoption and integration of evidence-based practices, interventions, and policies into regular use by healthcare professionals and organizations to improve health outcomes [40,41]. A significant emphasis is placed on the formulation of pragmatic costing tools, devising engagement strategies, and developing methods for contextual assessment. Additionally, the field incorporates rapid data collection strategies and technological tools for monitoring adaptations, as well as the creation of fidelity scales. These are all directed towards augmenting health equity and the practical application in clinical and community settings. Regarding capacity building, the focus extends to fostering research capacity among partners and practitioners. This involves assisting in leading grants, the development of research infrastructure, and enhancing skills pertinent to implementation processes. Moreover, there is a concerted effort to craft practitioner toolkits and tools for the iterative application of D&I science products. These are strategically designed for planning, adapting, and sustaining evidence-based practices in real-world environments, reinforcing the practical utility and reach of D&I science.

### Data collection

To optimize data collection within the ISC3 consortium, we developed a specialized data collection instrument primarily intended for pilot project investigators (PIs) who have concluded their projects. This instrument was designed for user-friendliness and visual appeal to promote efficient data submission by the pilots. It integrates essential elements from the TSBM to effectively encompass a range of impacts. (See example in supplementary materials). The data collection tool delineates specific data categories, organized as follows:

- ISC3 Project or Unit Identification: This section identifies the specific project or unit under the ISC3 consortium’s umbrella.
- Target Audience Definition: This segment aims to specify the intended or potential beneficiaries of the project’s deliverables. These beneficiaries may encompass clinical and public health leaders/administrators, policymakers, community stakeholders, and fellow researchers. Accurately identifying the audience is crucial for successfully disseminating and applying the project’s findings.
- Output and Partnership Documentation: This area details the outputs generated by the project or unit, which might include a variety of forms such as case studies, toolkits, dashboards, policy briefs, infographics, podcasts, and more. Additionally, it records any partnerships or collaborations fostered during the project. The emphasis here is on concrete deliverables, establishing or enhancing partnerships, and efforts in capacity building. Products that cater to the academic community, notably methodologic advancements and those targeting nonacademic audiences, hold particular importance.
- Domains of Benefits (from the TSBM Model): This requires the PIs to categorize the benefits of their project as per the TSBM model.
- Assessment of Demonstrated or Potential Impact: PIs detail their project’s actual or anticipated impact in this section.
- Support Requirements from ISC3: Here, PIs convey their needs for further developmental assistance to advance and disseminate their project outcomes effectively. They are also encouraged to propose a timeline that outlines when this support would be most advantageous.
- Projected Next Steps: This final section is where PIs delineate their planned future activities for their project or unit.

These categories were determined based on two primary goals. The first goal was to create an impact inventory for all ISC3 consortium projects, which is why we included categories such as Unit Identification, Output, Domain of Benefit, and Assessment of Benefit. The second goal was to identify potential audiences, leading to the addition of a Target Audience category. This was intended to inform a potential dissemination plan and storytelling for some of the outputs developed by the ISC3 consortium. Internally, we aimed to understand the next steps for our pilot projects and identify any dissemination roadblocks they might encounter. Therefore, we included the Next Steps and Support Required categories. A legend accompanies the tool to facilitate comprehension and elicit detailed responses, elucidating each category. Inputs for each domain were self-reported by the PIs in collaboration with other team members.

### Data analysis

The data collection tool was distributed across the seven centers. Participation was voluntary, as it was not a reporting requirement. We gave PIs a month to respond to the data collection tool, and we received responses from all seven centers, achieving 100% participation, with each center reporting more projects than the initial two or three projects requested. Notably, our TSBM tool adaptation was versatile enough to fit methods pilot studies, pilot projects, and even units such as the Community Incubator, which focuses on fostering connection with community partners. We aggregated data from seven centers and performed descriptive analyses focusing on three primary areas: TSBM domains, identified audiences, and the needs recognized by the pilot projects at each center. In analyzing the TSBM domains, our approach was to summarize the number of connections each project had with a given domain, treating each connection as a contribution. A single project could be linked to multiple domains.

We classified audiences based on the groups identified by the projects. Our developed categories included Public Health Practitioners, Primary Care Providers, D&I Researchers, General Researchers, Other Clinical Setting Audiences, Policymakers, Community Members, and Others.

The categorization of identified needs was similar. We sorted the requests from the projects into categories such as Tool Dissemination, Product Feedback, and Support for Early Career Investigators.

### Translating research findings

In selecting projects to highlight, we prioritized ensuring they resonated with our primary audience. This was crucial in our impact evaluation, especially in demonstrating the consortium’s overall impact to our funders. Therefore, we focused on projects that provided qualitative and quantitative evidence of their effect or, at the very least, presented a reasonable expectation of such impact. Our goal was to curate comprehensive impact outcomes to turn them into stories that would forge a meaningful connection with audiences other than researchers (e.g., practitioners, policymakers, funders). To this end, we presented our findings at an in-person meeting with our funders and other ISC3 collaborators, as well as during scientific conferences. For others looking to select impactful stories in their work, we suggest identifying the target audience, tailoring the presentation accordingly, and supplementing it with any necessary additional data.

## Results

Based on data from 48 ISC3 pilot studies, cores, or consortium activities across the seven research centers, we identified 84 distinct benefits. We did not collect data on whether the pilots or units planned these impacts from the beginning. Instead, as a consortium impact assessment, we collected the data retrospectively. Implementation science was the most commonly observed impact (43%), followed by community/public health (28%), clinical areas (18%), policy (8%), and economic aspects (2%). When we examined contributions per subdomain, we found that the most common contribution within the implementation science domain was in the capacity building subdomain (61%). In the clinical domain, the largest contribution was towards tools and products developed (62%), followed by procedures/guidelines (38%). In the community domain, we identified three subdomains: healthcare delivery activities (50%), health promotion (38%), and healthcare characteristics (13%). The policy domain saw contributions across three subdomains: advisory activities (45%), followed by an equal distribution between the impact of policies and legislation, and public health practices (27% each). Within the economic domain, the only subdomain that saw contributions was financial savings and benefits.

The second largest impact domain identified by the ISC3 consortium was community, reflecting our participatory and equitable approach. Involving community members is a crucial aspect of advancing the pursuit of health equity. While we acknowledge that involving community partners alone is not enough to eliminate health disparities, it does demonstrate that this was one of our top impact priorities.

Although the data reported here are aggregated, we provide an example to illustrate what we mean by impact in a domain. For instance, the Network Navigator Tool helped create linkages between clinics to share strategies for using evidence-based interventions [42]. Another contribution to the community domain of this project was to the subdomain of health education and community by developing new networks for prevention activities. The Network Navigator Tool also contributed to the capacity-building domain in implementation science by helping community partners develop their research infrastructure.

**Figure 2.**
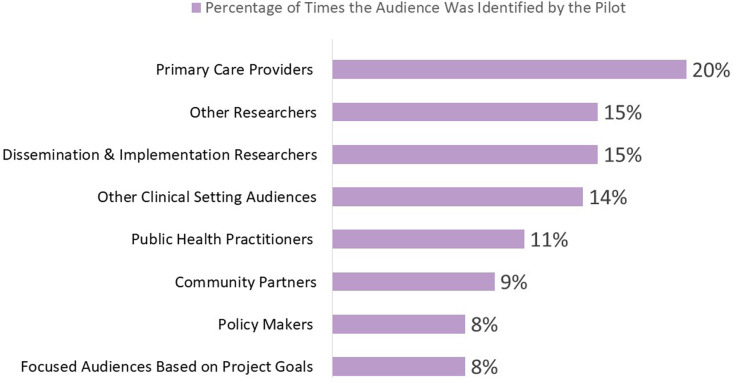
Audiences identified by pilots projects for impact dissemination.

**Figure 3.**
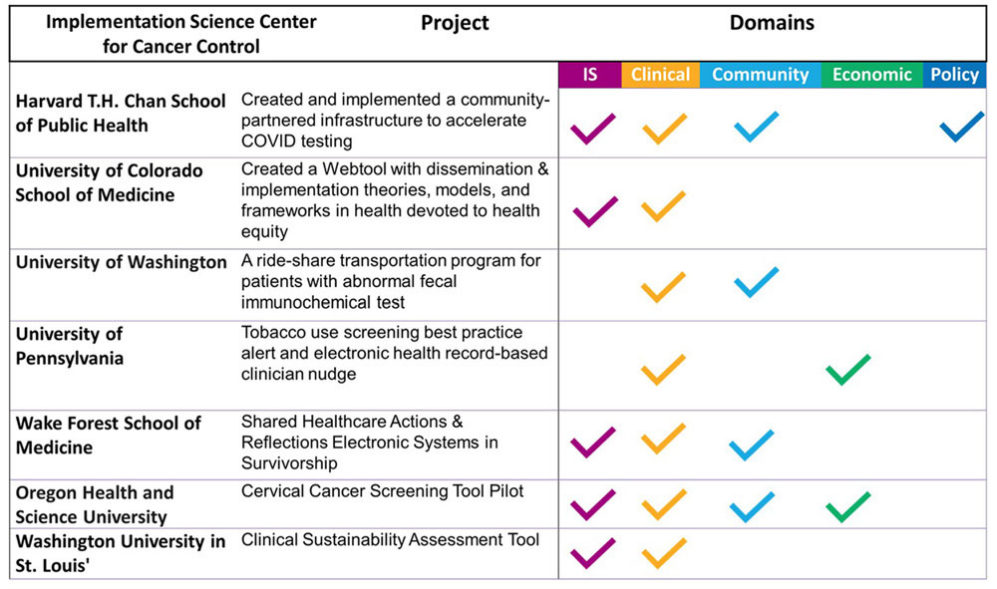
Impact examples across the implementation science center for cancer control consortium.

As shown in Figure 2, the audiences that benefited most frequently included primary care providers (20%), followed by D&I researchers (15%), other researchers (15%), audiences in other clinical settings (14%), public health practitioners (11%), community partners (9%), policymakers (8%), and some focused audiences that depended on project goals (8%). School personnel are an example of a focused audience. This audience was the focus of an ISC3 pilot study that examined Missouri school food services’ decision-making processes in applying and evaluating flexibility in nutrition standards for milk, whole grains, and sodium within the National School Lunch and Breakfast Programs [43]. The primary audiences identified for disseminating these findings were school food directors and industry food suppliers, aiming to provide them with insights and implications [44]. Examples of projects from each research center and their identified impact categories are shown in Figure 3.

A range of needs were identified to enhance the beneficial impacts of the initiatives. The survey of ISC3 centers revealed that 27% of the consortium members underscored the need for feedback on various products, such as web tools, toolkits, and clinical guidelines. A large focus was also placed on the dissemination of their work, with 21% seeking assistance in both website dissemination and the importance of increasing internal dissemination within the ISC3 consortium, 18% in the development of case studies and guidelines, and 6% in storytelling. Furthermore, 6% of the responses highlighted the necessity of supporting early career investigators, pointing to the need for investment in development opportunities for newly trained implementation researchers. The survey responses also illustrated a concerted effort towards integrating and addressing health equity within ISC3 activities, alongside effectively improving materials and methods to incorporate health equity theories, models, and frameworks.

**Figure 4.**
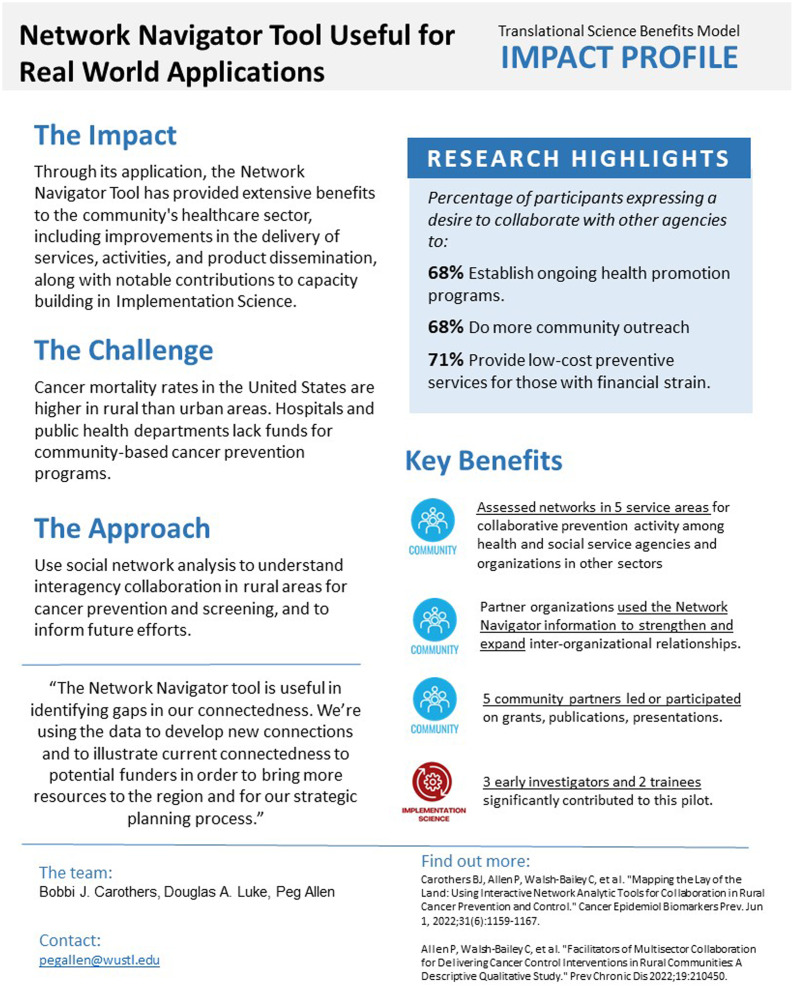
Network navigator tool: pilot impact profile example.

Our final step was disseminating our findings. The projects we selected to highlight were delivered during a presentation to our consortium colleagues and funders. These projects were selected based on the amount of data we had collected on their impact and how well they would resonate with our primary audience, our funders. For this purpose, we adapted the TSBM tools to create a compelling presentation, summarizing some of the impacts we observed in the ISC3 Consortium. An example of another dissemination product was developed using the TSBM Impact Brief, which allows researchers to tell the story of a project on one page (Figure 4). In this study, the team employed social network analysis to map informal networks for cancer prevention and control in rural communities [42]. Subsequently, a Network Navigator tool was developed from a survey involving informal multisectoral networks of 152 agencies. The team then analyzed and disseminated descriptive network statistics to rural agencies through infographics and interactive Network Navigator platforms. Furthermore, they sought feedback from agency staff regarding the network findings’ uses, usefulness, and impacts.

## Discussion

The ISC3 consortium’s application of the TSBM in a participatory framework is noteworthy in many ways. The model’s capacity to guide investigators in identifying and categorizing the real-world impacts of their projects across all TSBM domains is an important finding.

As mentioned, our consortium and research portfolio demonstrated impacts across the four TSBM domains: clinical, economic, community, and policy. When we compare the TSBM domains and subdomains of impact, several align with the National Cancer Plan’s goals of being health-centric and empowering [45]. For example, in the implementation science category, which had the most identified impacts, one of the subdomains includes tools and products to improve workflow development for evidence-based practices, along with other support to help implement high-value cancer prevention and control. This aligns with the health-centric goals of preventing cancer, detecting cancer early, and delivering optimal care.

Our second largest domain was community, which includes several subdomains such as community health services, health education and community resources, healthcare accessibility, healthcare delivery and quality, life expectancy, disease prevention, and public health practices. These subdomains capture the impact and fit within the goal of empowering communities, specifically to eliminate inequities and engage every person. Further information about our specific pilot projects is available on their respective websites [33–39].

Our impact findings are similar when compared against other methods focused on multi-pilot study assessments, establishing the utility of applying the TSBM. Our study builds on research using a variety of approaches to assess the impacts of research conducted mainly outside the United States. For example, Hanna et al. employed the REF to scrutinize the impact of 106 individual cancer trials through 46 case studies [46]. This in-depth analysis aimed to unravel the complex nature of impacts in cancer research, especially in policy influence, where trial findings were instrumental in shaping clinical guidelines. While distinct from ours in its reliance on case study analysis, their approach revealed similar trends in the less frequent economic impacts, echoing our observations.

Related examples in the literature show the application of the TSBM. For instance, Miovsky and colleagues used the TSBM to assess the impact of two CTSA-supported cohorts of COVID-19 studies [47]. Their focus on comparing the benefits of different COVID-19 projects conducted by campus-community partnerships (*N* = 6) or campus-only projects (*N* = 31) revealed differing impacts on clinical outcomes, community health, and economic benefits, enriching our understanding of various collaborative approaches. Unlike campus-only projects, where 26% reported clinical benefits, campus-community partnerships yielded no clinical benefits. However, campus-community partnerships were more effective in realizing community health (17% vs 10%) and economic benefits (17% vs 13%). A primary issue identified was the inability to verify 64% of self-reported benefits due to either misalignment of descriptions with selected benefit categories or insufficient detail for definitive verification. Similarly, in our analysis, we encountered challenges that required us to seek additional information or clarification from the pilot team regarding their reported impacts. This points out the limitation of self-reported impacts and emphasizes the need to take time to verify these impacts and develop methodologies to address verification challenges.

In another example, the TSBM was adapted to better track research and educational activities [48]. The main objectives were to enhance the model’s integration into practical applications and explore ways to expand the TSBM as a conceptual framework. The team devised a strategy to incorporate the TSBM across three key areas to broaden its application. First, they expanded the model’s use from individual research studies to encompass broader translational research programs, notably in workforce development. Second, TSBM’s frameworks were integrated into a new Duke CTSA database to track and evaluate the program’s activities and outcomes systematically. Third, the model was embedded into the pilot project application and review processes, ensuring its principles were applied from project initiation. This approach demonstrated the TSBM’s versatility and critical role in enhancing translational research and science practices. The challenge of differentiating between potential and demonstrated benefits was a common thread in our study and Duke’s, highlighting a universal challenge in impact assessment.

The comparative analysis of these diverse studies underscores the multifaceted nature of evaluating research impacts. Each method, whether focusing on case studies, structured data collection tools, or economic assessments, offers distinct insights and challenges. The TSBM has demonstrated versatility and utility across various contexts, from individual projects to broader consortium-based applications. However, a recurring challenge across these methods remains the differentiation between potential and demonstrated impacts, emphasizing the need for continuous refinement in impact evaluation approaches. Differing time horizons can be a challenge. For example, policymakers often seek impact data quickly, whereas the research process tends to be slow and deliberate [49]. This exploration not only highlights the strengths and limitations of current methods but also paves the way for future research in the field of impact assessment, encouraging the application, adaptation, and refinement of the TSBM and other frameworks mentioned as impact documentation tools.

Another important aspect is that the pilot project needs were mostly around dissemination, including storytelling, highlighting the importance of providing the resources to investigators to convert their research impact into compelling stories that resonate with their audiences. The products created by the projects will need to segment the identified audiences to determine which groups will benefit most from the products and tools developed. Audience segmentation helps in understanding who the ultimate target audience is. For example, social marketing uses demographics, motives, attitudes, benefits, barriers, and readiness to determine target audiences and the appropriate channels to reach them [50]. Moreover, segmentation involves asking thoughtful questions, learning from literature, interviewing key informants, and listening to the target audience [50]. Once audiences are identified, storytelling and narrative techniques can be powerful tools in making scientific insights accessible and relevant to decision-makers and the general public, highlighting the importance of framing narratives to enhance public engagement and demonstrate the practical impacts of research [51–53]. As our data showed, dissemination is a major need, and as researchers, we need to be intentional. It is crucial to consider the need for a transdisciplinary approach to add someone with expertise in communication to address nonacademic audiences.

The next step for this work would be to provide resources for each pilot to use the TSBM tools to create dissemination products tailored to the specific audiences identified. Our goal as a research consortium was to capture and communicate the impact of our research portfolio to other scholars, research partners, and funders. Using the TSBM tool to create case studies, impact profiles, or other products for each study will enhance the usefulness and potential impact of the dissemination products.

This study may be the first to use the TSBM to evaluate the collective impacts of a large, national-scale research consortium. While other models like the REF and the Payback Method have been used to assess institutional impact and multisite projects, the TSBM has not been applied in this context before.

## Conclusion

Efforts to translate scientific evidence into practice and policy are based on the idea that tangible benefits are associated with the increasing use of evidence-based interventions. However, the field has relied mainly on standard academic metrics to judge impact (e.g., publications, grants, scientific presentations). The TSBM, when applied using standardized methods, is a systematic way to more broadly document impact. Our summary of 48 pilot studies conducted across the ISC3 shows a wide range of impacts that are beginning to be realized. We anticipate some of these impacts will be sustained and magnified in the coming years.

We hope that others can use our data collection methods and develop creative ways of telling compelling stories about the impact of research. The D&I field needs to prioritize ways to more fully document and enhance implications for clinical and public health practice, policy and systems change, and health equity. Our approach may benefit those trying to document and capture the collective impacts of research consortiums.

## Supporting information

Cuevas Soulette et al. supplementary material Cuevas Soulette et al. supplementary material

## Supplementary material

The supplementary material for this article can be found at https://doi.org/10.1017/cts.2024.587.
