# Supplementary material for: Rethinking our future: Describing and enhancing the impacts of dissemination and implementation science for cancer prevention and control
Source: J Clin Transl Sci. 2024 Oct 10;8(1):e159. doi: 10.1017/cts.2024.587 (PMC11557277; doi:10.1017/cts.2024.587)
Supplement: Cuevas Soulette et al. supplementary material [file S2059866124005879sup001.docx]

**Supplementary Information: ISC3 Network TSBM data collection tool.**

| *ISC3 project (Include principal investigator/ lead)* | *Audience*^a^ | *Product(s)^b^* | *Domain of benefit^c^* | *Demonstrated or potential impact indicators^d^* | *Support needed/wanted from ISC3^e^* | *Next steps^f^* |
| --- | --- | --- | --- | --- | --- | --- |
|  |  |  |  |  |  |  |

**^a^Audience**: The audience is the group(s) to whom your product is being or could be disseminated and used. Audiences might include clinical leaders/administrators, public health leaders/administrators, policy makers, community members, and researchers.

**^b^Product (including partnerships)**: The product is the output from your project, your unit, or capacity building. Some examples include: case studies, tool kits, dashboards, policy briefs, infographics, and podcasts. Products can also involve the building of partnerships or relationships developed as a result of a project or unit. It is important to consider both the tangible products that you create and new/enhanced partnerships and capacity building since they often lead to impacts. Many methods advances are relevant mainly for scientific audiences (and should be reported)—we are primarily interested in products for audiences outside of academia.

**^c^Domain of benefit**: The domain is one of the five broad headings from the TSBM shown below, along with the sub-domain. You may note more than one domain for each product.

1. IMPLEMENTATION SCIENCE FIELD

*1a. Implementation Science Methods and Measures*

*1b. Capacity-Building*

1. CLINICAL

*2a. Procedures/Guidelines*

*2b. Tools and Products*

3. COMMUNITY

*3a. Health care delivery, health activities & products*

*3b. Health Care Characteristics*

*3c. Health Promotion*

1. ECONOMIC

*4a. Commercial Products*

*4b. Financial Savings and Benefits*

1. POLICY

*5a. Advisory Activities*

*5b. Policies & Legislation*

*5c. Public Health Practices*

**^d^Demonstrated or potential impact indicators**: The impact indicators can be drawn from the bulleted list in the attached document (”ISC3 Common Core Impact Indicators”). You should also note any impact indicators that are not on the attached list.

**^e^Developmental support from ISC3**: The types of support involve the help you need for further developing and disseminating the product you have listed. You should include information on the timing (e.g., ready for development now, ready for development in three months).

**^f^Next steps**: The next steps might include how to design the product for the communities of focus, feedback loops on how the product might change the research process, or how you might increase demand for the product as it is disseminated.
